# Endoscopic-Ultrasound-Guided Radiofrequency Ablation for Pancreatic Tumors

**DOI:** 10.3390/jcm14020495

**Published:** 2025-01-14

**Authors:** Chiara Coluccio, Stefania Cappetta, Giovanna Romagnoli, Valentina Di Giorgio, Paolo Giuffrida, Stefano Fabbri, Carlo Fabbri, Cecilia Binda

**Affiliations:** 1 Gastroenterology and Digestive Endoscopy Unit, Forlì-Cesena Hospitals, 47121 Romagna, Italy; 2 IRCCS Azienda Ospedaliero-Universitaria di Bologna, Policlinico S. Orsola, 40138 Bologna, Italy; 3 Department of Medical and Surgical Sciences, University of Bologna, 40138 Bologna, Italy

**Keywords:** endoscopic ultrasound, radiofrequency, ablation, locoregional therapy, pancreas

## Abstract

Endoscopic ultrasound (EUS)-guided radiofrequency ablation (RFA) is a promising minimally invasive technique for the treatment of pancreatic lesions. This review first focuses on the technical aspects in EUS-RFA: the procedure typically employs EUS probes with integrated radiofrequency electrodes, enabling accurate targeting and ablation of pancreatic lesions. Different types of RFA devices, monopolar and bipolar energy delivery systems, are discussed, along with considerations for optimal ablation, including energy settings, procedure time, and pre- and post-procedural management. This paper presents a comprehensive literature review of EUS-RFA applied to both solid and cystic pancreatic lesions, including functioning and non-functioning pancreatic neuroendocrine tumors (pNETs), pancreatic cystic lesions (PCLs), pancreatic ductal adenocarcinoma (PDAC), and pancreatic metastases (PMs), discussing current evidence on safety, efficacy, clinical outcomes, and adverse events (AEs). EUS-RFA is an emerging technique with expanding potential for the treatment of both benign and malignant conditions; however, further studies are needed to better define patient selection criteria, assess long-term benefits, and establish definitive indications for its use.

## 1. Introduction

The increase in diagnoses of solid and cystic lesions of the pancreas is substantial worldwide, due to the improved diagnostic performance of cross-sectional imaging machines and the aging population.

At present, surgery is the treatment of choice for most pancreatic lesions, both for malignant forms such as pancreatic ductal adenocarcinoma (PDAC) as well as for benign forms with non-negligible evolutionary potential or for symptomatic benign lesions; this is the case for neuroendocrine tumors (pNETs) and pancreatic cystic lesions (PCLs). Although in high-volume centers, mortality associated with pancreatic surgery has been reduced over the years, surgery-related complications are still very common (up to 50% of patients), with the most frequent and feared being postoperative pancreatic fistula (POPF), which has an incidence ranging from 3% to 45% in specialized centers [1,2]. In addition, many pancreatic lesions with a low risk of malignancy often require long-term surveillance, leading to increased healthcare costs.

In the last twenty years, the endoscopic ultrasound (EUS) technique has increasingly evolved in an interventional and therapeutic sense, expanding its initial purely diagnostic purpose.

EUS-guided radiofrequency ablation (EUS-RFA) is a novel minimally invasive technique for the treatment of pancreatic focal lesions, which produces a high-resolution, real-time image of the target area and ablates lesions through the application of thermal energy via radiofrequency. This results in irreversible cell injury, and simultaneous control supports high accuracy, minimizing the effect on surrounding healthy tissue [3].

Among the thermoablative technologies, RFA is one of the most widely used in the field of oncology (i.e., hepatocellular carcinoma); however, its application for the treatment of focal lesions of the pancreas is relatively new and not yet fully understood. In summary, a high-frequency alternating current generates high thermal energy (60–100 °C), and thus cells undergo protein denaturation, leading to coagulative necrosis [4]. Through a mechanism of indirect tissue damage, RFA also promotes the activation of antitumor immunity by remodeling the cancer microenvironment, inflammatory cytokines’ release, dendritic cells’ activation, and CD4+ and CD8+ T cells’ response [5].

The aim of this review was to collect literature data on the application of EUS-RFA, to both clarify technical aspects of the procedure itself, by grasping the common points and differences in the various published studies, and to gather information on all possible indications for EUS-RFA and the expected results from its employment in clinical practice.

## 2. Focus on the Technique

In 1999, Nahum Goldberg S. et al. first published the results of applying EUS-RFA with modified 19-gauge needle electrodes on healthy pancreatic tail tissue from 13 Yorkshire pigs using a transgastric approach, demonstrating that the method was technically feasible and safe [6].

Since then, the interest in this technique has gradually grown, leading to the production of dedicated devices and the publication of studies in animal models and later in humans. However, there is still no formal technical protocol accepted by scientific societies for EUS-RFA, and thus the technical details must be gathered from experts’ opinions developed in recent years.

The procedure is performed under deep sedation or general anesthesia with the patient positioned in left lateral decubitus. A linear-array echoendoscope with a 3.8 mm operator channel is advanced along the upper digestive tract to visualize the target lesion and, after exclusion of vascular structures by color Doppler imaging, the device is inserted into the lesion under EUS guidance with a transgastric or transduodenal approach (bulb or second part of the duodenum). The generator is turned on and the wattage set, and immediately afterwards, the endosonographer proceeds with ablation. Then, the tool can be repositioned in another part of the lesion to perform further ablation and the procedure repeated until the entire lesion is covered.

### 2.1. EUSRA™ Endoscopic UltraSound guided Radiofrequency Ablation electrode (TaeWoong Medical, Goyang-si, Gyeonggi-do, Republic of Korea)

#### 2.1.1. Description of Device

The EUSRA™ electrode is a modified 19 G needle for RFA consisting of a 140 cm flexible metal wire coated with an insulating cover, except for the exposed end portion of 5 mm, 7 mm, or 10 mm in length, and ending with a tapered, sharp tip capable of releasing energy in the form of heat (monopolar device).

The VIVA Combo™ RF Generator System (STARmed, TaeWoong Medical, Goyang-si, Gyeonggi-do, Republic of Korea), consisting of two components, is also required to carry out the procedure:

▪ VIVA Combo™ RF Generator, a current generator that operates at a frequency of 480 KHz, allowing the power setting to be varied from 0 to 200 watts, enables real-time monitoring of temperature (5–95 °C) and impendence (10–800 Ohms) of the tissue. It is connected via a cable to the upper end of the EUSRA™. Two grounding pads must be attached to the patient’s skin before the procedure.
▪ VIVA Pump is an internal cooling system that, through two tubes connected to the handle of the needle, allows cold saline solution (0 °C) to be pumped into the needle (inflow tube), which circulates inside and then is expelled warm through the second tube (outflow tube) into an external container. Circulation of cold saline solution inside the needle allows continuous cooling of the active needle tip, preventing tissue charring [7,8]. Tissue carbonization is indeed associated with increasing tissue impedance and reaching an ablation plateau with disruption of coagulative necrosis; hence, the cooling system should improve performance by providing ablation of a larger volume of tissue.

The ablated volume depends both on device and preset characteristics (wattage setting, length of active tip, and application time) and on intrinsic characteristics of the tissue (temperature and humidity). Barret et al. compared two ad hoc devices for EUS-RFA on the pancreas in a porcine model; the data collected suggest using EUSRA™ at 30 W for 15 s of application to achieve an ablation volume of 302 mm^3^, corresponding to an oval section of about 7 mm in diameter [9].

#### 2.1.2. Technical Features

EUSRA™ is positioned directly inside the target lesion, likewise a fine needle aspiration (FNA) needle. After setting up the generator, the operator can control the start and end of the ablation with a foot switch.

### 2.2. Habib™ EUS RFA Endoscopic Ultrasound Radiofrequency Ablation Catheter (EMcision Ltd., London, UK)

#### 2.2.1. Description of Device

The Habib™ EUS RFA is a “through-the-needle” device no longer commercially available since 2018; nonetheless, it deserves a mention as it has been used in several pioneering papers for EUS-RFA. The Habib™ EUS RFA is a flexible catheter with a diameter of 1 Fr (0.33 mm) and length of 220 cm; its distal end consists of an electrode with a length of 10 or 20 mm, and can be inserted through a 19 G or 22 G FNA needle. The probe is connected via an adapter cable to a regular electrosurgical generator with no cooling system. As a monopolar device, the attachment of a grounding pad on the patient is necessary.

#### 2.2.2. Technical Features

After proper visualization of the pancreatic gland, a 19 G or 22 G FNA needle is advanced within the target lesion in a deep location. The stylet is removed, and the Habib probe is cautiously advanced inside the needle until resistance is sensed. At this point, the needle is slowly retracted a few centimeters to expose the active tip of the catheter, to avoid contact between the electrode and the metal part of the needle, representing a delicate phase for the endoscopist in seeking to maintain the position.

### 2.3. Hybrid-Therm^®^ Probe HTP (ERBE, Tübingen, Germany)

#### Description of Device

Contrary to the description above, the HTP is a bipolar RF probe that combines cryotechnology with conventional thermoablation. The probe is fully covered with a protective tube 150 mm long; the stiff and pointed distal end is the electrically active part, with a diameter of 1.8 mm and a length of 20 mm. Connector cables for the current generator (VIO 300D, ERBE) and the internal carbon dioxide cooling system (ERBOKRYO CA System, ERBE) branch off from the proximal side of the handle. The wattage setting and gas pressure can be controlled independently. Integration of cryotechnology is intended to increase the effectiveness of RF by decreasing the power of the generator and thus reducing the risk of damage to the tissue.

As already mentioned, there is currently no standardization of the procedure and the setup of equipment. Therefore, many questions remain open in this regard.

### 2.4. Electrode Insertion and End of Ablation

Differences are reported in the literature regarding the criterion used for inserting the electrode within the lesion; some echoendoscopists place it directly in the center of the mass [10], while others position the echogenic tip at the far end of the lesion [11,12,13]. In another technique, the echogenic tip is inserted first in the distal right portion of the lesion, and then the process is repeated in the left portion [14]. To perform multiple applications, the needle can be retracted slightly to repeat the procedure on the same trajectory, or a new puncture can be made to insert the needle at a different angle [15,16].

In most early studies, the RFA procedure was considered complete when echogenic bubbles began to appear around the needle tip and progressively covered the entire lesion after one or more applications. It is currently assumed that to increase the accuracy of thermoablation, another useful element to evaluate at this stage is the bioimpedance, continuously tracked by the generator, because a sudden rise in impedance may be an indicator of early tissue charring. Scopelliti et al. [8] considered a value of 500 Ohms adequate to end the ablation; concurrently, Barthet et al. [17] stopped the procedure when the impedance settled in the range of 100–500 Ohms and white bubbles appeared on the screen.

### 2.5. Power Setting and Application Time

A variety of wattage settings have been used on the pancreas in recent studies, both due to the technical specifications of the devices and the decisions of the operators.

The Habib™ EUS RFA has been described with a power range from 5 W to 25 W [11]; however, the most widely used is 10 W or ERBE SOFT COAG mode effect 4, with an application time between 90 and 120 s.

Using an early prototype 19 G EUSRA™, Lakhtakia et al. [12] published the results of three cases of patients with insulinoma (maximum diameter 22 mm) treated with EUS-RFA, using a power setting of 50 W for an application time of 10–15 s and a mean number of applications of 5. A similar technique was used in Song et al. on six patients with PDAC with an average diameter of 38 mm [14]. Differently, Crinò et al. [7] proposed a technical approach with EUSRA™, involving a wattage setting of 30 W for a mean application time of 50 s (not determined in advance) and a mean number of applications of 1.5 in seven cases of PDAC and one case of metastasis from clear-cell renal cell carcinoma. Reducing the power and extending the application time allowed the ablated volume to be increased as a result of slower thermal diffusion, thus reducing the number of applications and the need to reinsert the electrode into the tissue.

A recent ex vivo study on RFA conducted on 15 fresh surgical samples of patients with PDAC treated with neoadjuvant therapy failed to establish a correlation between power setting group (10 W, 30 W, and 50 W) and the mean short-axis diameter of coagulative necrosis, as determined by two blind expert pathologists [18].

In contrast, the Hybrid-Therm^®^ Probe was set at 18 W power with a carbon dioxide gas pressure of 650 psi and an application time in the range of 240 to 480 s on PDAC lesions from 20 mm to more than 30 mm in diameter [19,20].

## 3. Pre- and Post-Procedural Issues

### 3.1. Antibiotic Use and Acute Post-Ablation Pancreatitis Prophylaxis

#### 3.1.1. Antibiotic Prophylaxis and Rectal Non-Steroidal Anti-Inflammatory Drugs (NSAIDs)

Evidence on the usefulness of antibiotic prophylaxis in therapeutic EUS, particularly in EUS-RFA, is limited. At this juncture, the lack of a clear protocol has left the choice up to the operators; in most of the papers, the administration is reported of peri-procedural antibiotic prophylaxis with broad-spectrum antibiotics (ceftriaxone, amoxicillin/clavulanic acid). Barthet et al. [21] had not planned on using any prophylaxis; however, two moderate–severe post-procedural adverse events (AEs) occurred in the first two patients treated: an acute pancreatitis with early infected necrosis and a small bowel perforation with retroperitoneal fluid collection requiring surgery. These two cases led to the modification of the study protocol to include the administration of antibiotic prophylaxis and rectal diclofenac, producing a significant decrease in adverse events (from 13.79% to 7.4%).

Another issue concerns the use of NSAIDs as a preventive measure for acute post-ablation pancreatitis, following the recommendations for acute post-ERCP pancreatitis [22]. The largest multicenter retrospective analysis we have available [23], reporting data from 116 EUS-RFA sessions, showed that antibiotic prophylaxis was administered in 85.3% of cases and that rectal NSAIDs were administered in 88.8%. This study attempted to identify risk factors for EUS-RFA-related adverse events by univariate and multivariate analysis; with the limitations and potential bias of a retrospective study, the analyses failed to identify a potential protective action of antibiotic prophylaxis and rectal NSAIDs

#### 3.1.2. Placement of Pancreatic Stent

The role of prophylactic placement of a pancreatic stent for lesions close to the main pancreatic duct (MPD) to prevent post-ablation pancreatitis remains controversial due to the procedure’s inherent risk of pancreatitis. In fact, although in most cases, post-ERCP pancreatitis is mild, its overall incidence ranges from 3.5% to 9.7% and increases to about 15% in high-risk patients [24].

Therefore, there is debate about the minimum distance from the MPD for RFA to be considered safe, beyond which the benefits of pancreatic stent placement outweigh the risks. In this case, the French study showed a significant increase in the incidence of AEs (OR, 4.1) in lesions in close proximity to the MPD (≤1 mm) [23]. Another large multicenter retrospective analysis by Crinò et al. [25] showed that in eight of nine cases of post-RFA acute pancreatitis, the distance between the lesion and the MPD was ≤2 mm, and although it did not reach statistical significance in a univariate analysis of risk factors for AEs, it remains an indispensable preprocedural parameter.

### 3.2. Fluid Component Suction Before EUS-RFA on PCLs and Cystic pNETs

In PCLs undergoing EUS-RFA, operators frequently reported making the choice to aspirate the liquid component using an FNA needle before the ablation, preserving only a thin layer of fluid. The purpose would be to reduce the overall volume of the lesion and avoid excessive application of current to the liquid component, thus reducing the risk of thermal damage and post-procedural infection [26]. However, it remains a debated practice due to the increased procedural time, number of passes and punctures, and also the potential reduced visibility of mural nodules and enhanced septa.

### 3.3. Role of Contrast Harmonic EUS (CH-EUS) and Follow-Up

CH-EUS has emerged as an evidence-based auxiliary diagnostic tool to optimize the characterization of solid lesions, improve the staging of malignancies, and target tissue acquisition. Recently, there has been a growing interest in the potential impact of CH-EUS in interventional procedures, including EUS-guided tumor ablation. Choi et al. [27] investigated its use to assist ablation, providing a delineated real-time visualization of the vascular lesion pattern, as well as its application in post-procedural monitoring. Technical success was achieved in all 19 patients (14 pNETs, 2 PCLs, 2 adrenal adenomas, and 1 adrenal metastasis from hepatocellular carcinoma); after approximately one week from the first RFA session, all patients underwent CH-EUS to check the treatment response, and 12 patients had residual tumor and therefore underwent further RFA sessions. At the one-year follow-up, 68.4% (13 patients) had a complete radiological response (CR). Borrelli de Andreis et al. [28] proposed CH-EUS-assisted RFA in 10 patients with insulinomas, injecting a new contrast bolus at the end of the ablation to verify vascularity, so that further RFA applications could be performed in the same session. At the 3-month follow-up, all patients had CR, and up to the end of follow-up (average 19.5 months), complete regression of symptoms was confirmed.

**Figure 1:**
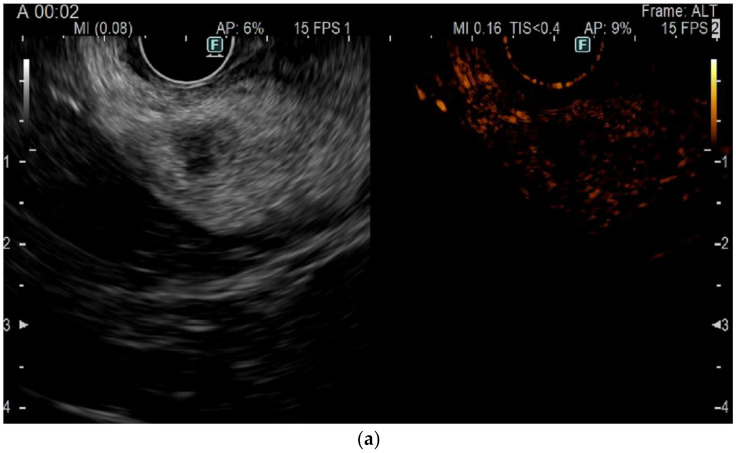

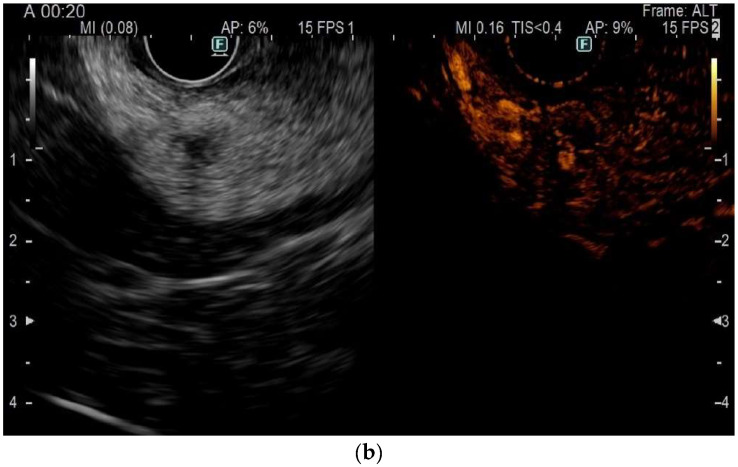
CH-EUS six-month follow-up in an asymptomatic patient after EUS-RFA on an insulinoma of the uncinate. (**a**) Time 0 corresponding to the injection of SonoVue^®^, (**b**) arterial phase with suspicion of millimetric residual disease, hypervascular in the image.

The timing of post-ablation surveillance with EUS or a CT scan or MRI is not supported by evidence; it could be useful to perform a first close work-up, within 1–3 months, to assess whether more RFA sessions are needed (see Figure 1).

## 4. EUS-RFA in Pancreatic Tumors

EUS-RFA is rapidly emerging as a cutting-edge technique in the endoscopic community, offering a minimally invasive solution applicable to a variety of pancreatic conditions. The best indications for the technique are still being studied, as are the characteristics of the ideal patient. The developing indications, characteristics of enrolled patients in the current literature, and mean lesion sizes are summarized in Table 1.

The most encouraging results come from the small (maximum size 20 mm) pNETs, particularly from the secerning variant. In contrast, the application of EUS-RFA in PCLs and PDAC is still at an early stage of study. As the field is continuing to evolve, the following section will delve into the different types of pancreatic lesions, solid and cystic, where EUS-RFA is gaining increasing attention for its potential application.

### 4.1. EUS-RFA in pNETs

pNETs can be both functional and non-functional. Functional pNETs (F-pNETs) are marked by a clinical syndrome related to abnormal levels of biologically activated hormones secreted by the tumor mass. On the other hand, non-functional pNETs (NF-pNETs) secrete hormone variants that are inactive or at low levels, and hence they are not associated with symptoms.

#### 4.1.1. F-pNETs

The most common digestive functional NETs are insulinomas and gastrinomas; the first is almost exclusively pancreatic, with an incidence of up to 32 cases 10^6^ of inhabitants/year and malignant in less than 10% of cases, while the latter is pancreatic in 25% of the cases, with an overall incidence of up to 21 cases 10^6^ of inhabitants/year and malignant in 60–90% of cases [29,30].

Currently, the treatment of choice for localized pancreatic insulinomas and gastrinomas is surgical resection. The knowledge of EUS-RFA on gastrinomas is limited to very few reports and with questionable clinical success, so its use should be limited to a trial setting. Otherwise, for insulinomas, the European Neuroendocrine Tumor Society (ENETS) has recently published a paper in which EUS-RFA is indicated as a possible treatment for insulinomas ≤2 cm in patients unfit for surgery in experienced centers, although with a low grade of recommendation [31].

Most of the available literature (reported in Table 2) on EUS-RFA in F-pNETs is related to case reports and case series with a small number of treated lesions. The first experience with EUSRA™ is that of Lakhtakia et al. [12], with a published case series of three symptomatic insulinomas successfully treated in a single session of EUS-RFA (mean size 17.3 mm) and with a mean number of applications of five. No post-procedural adverse events occurred, and in all three cases, a rapid improvement of hypoglycemia was observed, with glycemic values settling into a normal range within 24 h after the treatment.

**Figure 2:**
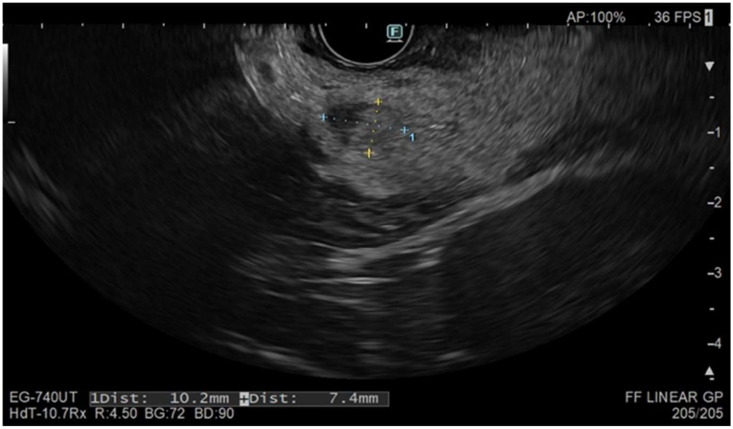
Six-month follow-up in an asymptomatic patient after EUS-RFA on an insulinoma of the uncinate: oval-shaped area with blurred margins and inhomogeneous hypo- and hyperechogenic structure.

In the same year, Waung et al. [32] also published a case report of an insulinoma (tumor size 18 mm) successfully treated with Habib™ EUS RFA following three sessions of RFA. Choi et al. in 2018 published a prospective study on EUS-RFA treatment of PCLs and pNETs, where a 12 mm insulinoma of the pancreatic head was successfully treated at 13 months of follow-up; however, in a 2020 study update, the same patient had experienced a recurrence at month 19 of follow-up [13,27]. To our knowledge, the largest case series is that presented by Crinò et al. [25] in a retrospective multicenter analysis collecting data from 89 patients with insulinoma who underwent EUS-RFA. In this study, the EUS-guided technique was feasible and safe, with a much lower rate of adverse events (AEs) compared to the control group undergoing surgery (procedure-related AEs: 18% vs. 61.8%). Of 89 patients, 16.9% experienced a recurrence of symptoms within one year after the procedure, and yet most of them were effectively treated with a new session of thermoablation (see Figure 2).

When comparing the papers, we found that most of the adverse effects related to EUS-RFA were mild to moderate (see Section 5). The only reported case of post-EUS-RFA death was that of a 97-year-old patient with multiple comorbidities, who developed an infected retrogastric collection; the patient refused further invasive interventions, so the collection was not drained and the patient died despite the best supportive care [33].

EUS-RFA is feasible and safe; it could represent a breakthrough in the treatment of F-pNETs, and promising data are reported in the literature. A recently published systematic review and meta-analysis that analyzed the results of nineteen studies with 101 F-pNETs showed a clinical success rate of 95.1%, with an overall AE rate of 17.8% [34]. Nevertheless, prospective randomized controlled trials with longer follow-ups are needed to confirm these records and to assess whether refinement of the endoscopic technique over the years will result in a reduction in the number of recurrences.

**Table 2:** Results from studies reporting EUS-RFA on F-pNETs *.

Author, Year	Study Design	N. of Patients/N. of F-pNET	Tumor Size (Mean, mm) and Location **	Device	N. of RFA Sessions/Lesion **	Mean Time of Application (s)/Mean N. of Applications **	Technical Success (%) **	Follow-Up (Months) **	Clinical Success (%) **
**Lesmana [35], 2024**	Case series	3/3	27.7 Head: 2 Body: 1	EUSRA™, TaeWoong (30–40 W)	NR	10–50/15	100	6–12	100
**Biermann [36], 2024**	Case series	3/3	14 Head: 2 Tail: 1	EUSRA™, TaeWoong (20 W)	1	NR/6.7	100	12.7	100
**Napoléon [23], 2023**	Retrospective, multicenter	16/16	15 NR	EUSRA™, TaeWoong (50 W)	1.12	25/3	97	13	87.5
**Crinò [25],** **2023**	Retrospective, multicenter	89/89	13.4 Head: 34 Body: 39 Tail: 16	EUSRA™, TaeWoong (10–50 W)	1.12	NR/3.2	100	23	95.5
**Borrelli de Andreis [28],** **2023**	Retrospective, monocenter	10/10	11.9 Head: 3 Body: 3 Tail: 4	EUSRA™, TaeWoong (25–50 W)	1.1	10/6.6	100	19.5	100
**Gugger [37],** **2023**	Case report	1/1 (somatostatinoma)	9 Tail	EUSRA™, TaeWoong (10 W)	1	NR/3	100	10	100
**Rizzatti [38],** **2023**	Prospective, multicenter	30/30	12.1 Head: 15 Body: 10 Tail: 5	EUSRA™, TaeWoong (50 W)	1.16	NR	100	12	100
**Figueiredo Ferreira [39],** **2022**	Prospective, multicenter	13/13	14.4 NR	EUSRA™, TaeWoong (50 W)	1	NR/3	100	12	100
**Marx [33],** **2022**	Prospective, multicenter	7/7	13.3 Head: 1 Body: 6	EUSRA™, TaeWoong (50 W)	1	NR/4	100	21	85.7
**Chang [40],** **2022**	Case report	1/1	12 Head	EUSRA™, TaeWoong (50 W)	1	10/2	100	18	100
**Rossi [41],** **2022**	Prospective, monocenter	3/3	16 Head: 3 Body: 3 Tail: 4	EUSRA™, TaeWoong (30 W)	1	11.8/3.3	100	22	100
**Younis [42],** **2022**	Prospective, monocenter	1/1	8 Tail	EUSRA™, TaeWoong (50 W)	1	NR/6	100	7	100
**Nabi [43],** **2022**	Retrospective, monocenter	12/15	17 Head: 6 Body: 8 Tail: 1	EUSRA™, TaeWoong	NR	NR	100	41	100
**De Nucci [44],** **2020**	Prospective, monocenter	5/5	12.8 Body: 3 Tail: 2	EUSRA™, TaeWoong (20 W)	1	15–25/2.2	100	12	100
**Lakhtakia [45],** **2020**	Prospective, monocenter	10/13	11.5 NR	EUSRA™, TaeWoong	NR	NR	100	10–64	100
**Kluz [46],** **2020**	Case report	1/1	9 Head	EUSRA™, TaeWoong (50 W)	1	10/3	100	NR	NR
**Furnica [47],** **2020**	Retrospective, monocenter	4/4	12 Head: 2 Body: 1 Tail: 1	EUSRA™, TaeWoong (50 W)	1	NR/2	100	22	100
**Jonica [48],** **2020**	Case report	1/1	22 Body	EUSRA™, TaeWoong (30 W)	1	20/5	100	6	100
**Borwn [49],** **2020**	Case report	1/1	18 Head	EUSRA™, TaeWoong (20 W)	1	15/NR	100	8	100
**Oleinikov [50],** **2019**	Retrospective, multicenter	7/9	14.8 Head: 7 Body: 2	EUSRA™, TaeWoong (10–50 W)	0.78	5–12/3–10	100	9.7	100
**Kandula [51],** **2019**	Case report	1/1	17 Head	NR	1	NR	100	6	100
**Choi [13],** **2018**	Prospective, monocenter	1/1	12 Head	EUSRA™, TaeWoong (50 W)	1	NR/3	100	13	100
**Gueneau de Mussy [52],** **2018**	Case report	1/1	12 Body	EUSRA™, TaeWoong	1	NR	100	2	100
**Thosani [53],** **2018**	Retrospective, multicenter	3/3 (including 1 VIPoma)	23 NR	NR	1.6	NR/4.6	100	5	100
**Goyal [54],** **2017**	Case series	1/1	7.5 Body	Habib™ EUS RFA (10 W)	1	120/3–5	100	NR	100
**Bas-Cutrina [15],** **2017**	Case report	1/1	10 Body	Habib™ EUS RFA (10 W)	1	120/3	100	10	100
**Waung [32],** **2016**	Case report	1/1	18 Head	Habib™ EUS RFA (10 W)	3	93.6/8.3	100	10	100
**Lakhtakia [12],** **2016**	Case series	3/3	17.3 Head: 3	EUSRA™, TaeWoong (50 W)	1	12.5/5	100	12	100

* Table legend: N. of RFA sessions/lesion expresses the ratio of the number of total procedures to the total number of pancreatic lesions. If equal to 1, the number of procedures is equal to the number of lesions; if >1, it indicates that some lesions are subjected to more than one session; if <1, it indicates that more lesions in the same patient are treated in the same session. Mean time of application (s) indicates the mean time per single application, expressed in seconds, to achieve the desired effect from ablation. Mean N. of applications indicates the mean number of applications needed to obtain complete treatment of the mass. NR: not reported. ** In studies involving more than one type of pancreatic lesion, the overall data were reported when it was not possible to extract the specific data per lesion group.

#### 4.1.2. NF-pNETs

The majority of pNETs, ranging from 60% to 90%, are non-functional [30]. Compared to their functional counterparts, NF-pNETs locate more frequently at the level of the pancreatic head and appear larger at diagnosis, with a more aggressive histopathology and more advanced stage of disease (like lymph node involvement or liver metastases) [55]. The gold standard for treatment is once again surgery, but with a non-negligible complication rate.

Due to the technological advancement of imaging techniques, the incidental diagnosis of pNETs is becoming increasingly frequent. These lesions are not biologically homogeneous; indeed, small and well differentiated ones often have an indolent behavior, with a low risk of malignant progression. While for NF-pNETs ≥ 2 cm, surgery represents the first-choice treatment [56], the best approach for NF-pNETs ≤ 2 cm remains controversial, as it is increasingly challenging to justify the risks of major surgery in these cases, with practitioners leaning towards active surveillance [57]. In this context, EUS-RFA could have a role as an alternative treatment; however, studies in the literature are scarce, restricted to a small number of patients, and not randomized. Another limitation is the difficult comparability of the results, due to different interpretations of the concept of a ‘radiological response’ among authors: univocally CR corresponds to 100% disappearance/necrosis of the lesion, demonstrated by an imaging technique; however, the definitions of a ‘Partial Response’ (PR) and ‘No Response’ (NoR) are variable. According to Barthet et al. [17,21], Figueiredo Ferreira et al. [39], and Younis et al. [42], a reduction of between 50% and 100% in the maximum diameter corresponds to a PR and a reduction <50% refers to NoR; according to Napoléon et al. [23], a PR corresponds to a reduction in the lesion volume of between 75% and 95% and NoR to a reduction <75%. Other authors, such as Marx et al. [58] and Choi et al. [13,27], do not give a quantitative definition of the radiological response, referring generally to the absence or persistence of enhancing tissue on the tumor site. This limitation is also found in studies on PCLs and PDAC. Among the most relevant papers, the multicenter prospective study by Marx et al. achieved a 92.6% CR in 27 NF-G1-pNETs at 15.7 months of follow-up, with two patients requiring more than one session to obtain this result; four patients developed acute post-procedural pancreatitis, requiring endoscopic or surgical maneuvers in three cases (see paragraph 8) [58]. Rizzatti et al. [38] published data on 32 NF-pNETs (median size 16.8 mm) successfully treated with single or multiple sessions of EUS-RFA with a 100% CR one year after the procedure and reported only mild AEs. In the retrospective analysis by Napoléon et al. [23], a CR of 71.7% was reported for 48 NF-pNETs (mostly graded G1) at 13 months of follow-up.

The longest follow-up was reported by Barthet et al. (45.6 months on average), where 12 of 14 NF-pNETs went on to reach CR at the 1-year follow-up, although 1 of these had a late recurrence at the 3-year follow-up. The other 2 of 14 experienced NoR at the 1-year follow-up: one evolved into metastatic disease, while the other had disappeared on both a CT scan and EUS at the 53-month follow-up. Thus, at 3 years, 85.6% achieved a CR (12 of 14 lesions) [17,21].

The literature data on EUS-RFA of NF-pNETs are summarized in Table 3.

### 4.2. EUS-RFA in PCLs

The detection of PCLs is increasingly common, mostly in completely asymptomatic individuals, due to the spread of cross-sectional imaging techniques and the aging population [61]. PCLs are heterogeneous, with different biochemical and histopathological features, and are classified as serous cystic neoplasms (SCNs), mucinous cystic neoplasms (MCNs), intraductal papillary mucinous neoplasms (IPMNs), and solid pseudopapillary tumors (SPTs). The risk of malignancy is widely variable in cystic panels: the evolutionary potential is considered negligible in SCNs, while it becomes moderate–high for SPTs and MCNs; namely, the probability of finding high-grade dysplasia (HGD) or invasive carcinoma (IC) in mucinous masses is associated with cyst-related factors such as a large size, presence of an enhanced solid component, duct dilatation, and patient-related factors such as the presence of symptoms and CA19-9 elevation [62]. These hazard attributes are also encountered in the risk stratification of IPMNs, classified as high-risk stigmata (HRS) and worrisome features (WFs). IPMNs have the widest variability in malignant evolution, ranging from the lowest risk of finding HGD/IC in brunch duct IPMNs (BD-IPMNs) without HRS and WF, to the highest risk in main duct IPMNs (MD-IPMNs), as demonstrated by histopathological samples [63].

The dramatic increase in diagnoses in comparison to the relative low degree of malignant transformation highlights the importance of an accurate characterization of cystic lesions to find a risk–benefit compromise between surveillance and treatment. In recent years, efforts have been made to find alternatives to surgery, such as less invasive local therapies for PCLs bearing a high risk potential for malignancy or that are symptomatic, ranging from EUS-guided ethanol ablation to EUS-RFA and EUS-fine needle injection (FNI). Despite its clinical relevance, only a few studies of EUS-RFA on PCLs are available, mainly case reports and small case series (literature data reported in Table 4), with different criteria for defining a radiological response, which limits the comparability of the results, as already mentioned for non-functional pNETs. The experience of Barthet et al. [17,21] was reported on 16 IPMNs and 1 MCN, with a total of 12 mural nodules undergoing RFA with EUSRA™: 40% and 26.7% of the subjects achieved CR and PR, respectively, with a mean follow-up of 42.6 months and all 12 mural nodules having disappeared. The most severe AE was perforation of a jejunal loop, which was managed surgically. Younis et al. and Napoléon et al. reported similar results on 11 and 5 PCLs, with a CR in around 60% and average follow-up of 13 months [23,42].

Conversely, none of the 13 SCNs reported by Oh et al. [26] reached a CR at the end of the follow-up, and five patients had NoR (defined as a reduction of less than 30% in longest diameter). An assumption is that the microcystic, honeycomb appearance of serous neoplasms may have hampered heat diffusion during ablation, thus affecting the outcome.

Rigorous studies and longer follow-ups are needed to understand the best indication of this minimally invasive technique in the context of pancreatic cystic lesions. For example, it is at least debatable that some studies have included SCNs, given their negligible malignant potential. Accordingly, so far, the only possible indication in this group of lesions is obtaining a sufficient cytovolumetric reduction in large cystic masses causing compression, with the aim of regressing symptoms and avoiding surgery, though existing studies have not focused on clinical aspects. Furthermore, the role of EUS-RFA is still vague for lesions with a higher risk of malignancy, such as MCNs, solid pseudopapillary tumors (SPTs), or IPMNs with worrisome features and high-risk stigmata; despite encouraging results on the ablation of mural nodules, solid data on late recurrences in CR and disease progression in PR are lacking.

### 4.3. EUS-RFA in PDAC

PDAC is the most frequent histotype of pancreatic cancer (over 80% of cases) and statistics report an increasing incidence trend in both males and females. Moreover, PDAC still holds primacy in terms of aggressiveness, with a 5-year relative survival rate of 13% [64]. The gold-standard treatment with curative intent is surgical resection plus perioperative chemotherapy, but less than 20% of lesions are resectable at diagnosis [65].

As with other solid tumors, in recent years, interest has arisen in local PDAC therapy in the context of a multimodal therapeutic strategy, with the aim of locally controlling the disease, reducing symptoms, and increasing survival. EUS-RFA has been proposed as a local ablative approach to decelerate tumor progression by promoting antitumor immunity and facilitating the penetration of chemotherapeutic drugs.

The first evidence of the application of EUS-RFA on PDAC in vivo is the Italian–German feasibility study [19] conducted on 22 individuals with locally advanced PDAC, using a prototype of the Hybrid-Therm^®^ Probe HTP (ERBE, Germany). The technical success of EUS-RFA was 72.8%, as stable probe insertion was not possible in six patients due to the stiffness of the tumor tissue and an infiltrated gastrointestinal wall. No survival benefit was observed (see Table 5). A randomized controlled phase II trial [20] compared the efficacy of ablative treatment with Hybrid-Therm^®^ Probe in combination with chemotherapy versus chemotherapy alone in the management of patients with locally advanced and borderline resectable PDAC; however, both due to the restricted inclusion criteria and the manufacturer’s withdrawal of the HTP probe, enrolment was difficult and the trial had to be stopped early: an intention-to-treat analysis was performed on 17 patients for the study group and on 20 for the control group. Progression-free survival (PFS) and volumetric reduction of the tumor mass at 6 months were slightly better for the study group, although without statistical significance; no relevant differences emerged in either surgical resection rate or OS. Oh et al. [66] reported the results of 22 patients with locally advanced and metastatic PDAC who underwent EUS-RFA in combination with gemcitabine-based chemotherapy before and after ablation: OS was 24.03 months with a PFS of 16.37 months, and when analyzing the subgroup of metastatic patients, the OS was reduced to 15.05 months. These results appear encouraging compared to what has been reported in previous studies; this could be explained by the fact that the EUS-RFA procedure was performed repeatedly if deemed unsatisfactory, for a total of 107 RFA sessions (a mean of 5 per patient), and the number of sessions correlated with PFS.

Currently, the literature on EUS-RFA in pancreatic cancer is rather scarce, most of the papers are small feasibility studies, data on the impact of this treatment on patient survival are often lacking, and those collected so far are not encouraging, with rare exceptions. Randomized controlled, multicenter trials are needed to establish whether EUS-RFA can really play a role in the multimodal treatment of PDAC and which patient groups can really benefit from it; currently, the involvement of patients with metastases is at least disputable.

### 4.4. EUS-RFA in Pancreatic Metastases from Renal Cell Carcinoma (mRCC) and Other Pancreatic Metastases (PMs)

The pancreas is rarely the site of metastatic localization of primary tumors of other origin. Clear-cell renal cell carcinoma is the neoplasm that most frequently metastasizes to the pancreas; the most frequent sites of secondarism for RCC are the lungs, bones, lymph nodes, and brain, although it may infrequently associate with glandular metastases (pancreas, breast, thyroid, and parathyroid). The literature suggests that glandular metastases, especially pancreatic metastases, have a more favorable prognosis than other localization sites [76,77]. Focal treatment is the treatment of choice in oligometastatic diseases, and surgery is the gold standard for pancreatic metastases.

A number of studies have proposed the use of EUS-RFA in pancreatic metastases as an alternative to surgery; however, data are still rather limited and the results are conflicting (see Table 6). Biasutto et al. [78] published the results of four pancreatic metastases from RCC undergoing EUS-RFA, showing that the procedure was feasible and safe in all three patients involved. At imaging control, an area of necrosis was confirmed in the treated sites; however, no clear data on the radiological response were given. Later, Chanez et al. [79] published one of the most relevant reports of EUS-RFA applied to pancreatic metastases from RCC: 21 lesions underwent 26 sessions of EUS-RFA. Two patients developed severe adverse events, which were managed with hospitalization and intervention; at the end of follow-up (mean follow-up of 27.7 months), all patients were alive. At radiological follow-up at 12 months, a complete response and a partial response (defined as regression of 30% or more of the tumor contrast uptake) were obtained in 40% and 33.3% of lesions, respectively. Similar findings were also reported by Napoléon et al. [23] out of 23 pancreatic metastases submitted to EUS-RFA. On the contrary, Figueiredo Ferreira et al. [39] showed completely different results in nine mRCC lesions and one metastatic lesion from lung cancer. In this case, there was failure in all renal metastases, and the only CR was seen in the lesion of lung origin. The authors speculated that such different results may be explained by both the different criteria used in defining the disease response and by the different disease stages and uses of systemic therapy.

## 5. AEs in EUS-RFA

The pancreas is a highly thermosensitive organ, and for many years, this has limited the application of radiofrequency ablation techniques in pancreatic lesions due to the risk of thermal damage of pancreatic tissue and nearby anatomical structures (wall of the GI tract, common bile duct, blood vessels). The few available studies on intraoperative radiofrequency ablation do not show reassuring results in terms of safety, with high rates of complications and post-procedural mortality [81,82]. The advent of EUS has opened a new door for radiofrequency ablation of pancreatic neoplasms, as real-time visualization of the lesion potentially minimizes the risk of thermal damage to healthy pancreatic tissue and adjacent structures.

After pooling the data from the studies presented above, the AEs were compiled (Table 7) and divided into classes of severity according to the Classification for Adverse Events GastRointEstinal Endoscopy (AGREE) [83]. AGREE classes I and II refer to mild–moderate adverse events that result in a prolonged hospitalization of the patient with conservative therapeutic management (e.g., administration of drugs such as antiemetics, antipyretics, analgesics, antibiotics, and antithrombotics, blood transfusion or blood products). AGREE classes III and IV refer to severe AEs requiring endoscopic or surgical intervention or admission of the patient to the intensive care unit. Class V implies the patient’s death.

We have data on post-treatment AEs, with EUS-RFA in 206 lesions of F-pNETs and 109 lesions of NF-pNETs, and with a mean number of sessions/lesion of 1.1 equal to the weighted value (w.v.), for a total of 325.9 sessions of EUS-RFA in 315 pNETs. Data from three papers [23,39,53] were excluded, as it is not possible to determine in which patients the reported AEs occurred (in the table, shown as Not specified). In 325.9 EUS-RFA sessions, 58 adverse events occurred, with a total AE rate of 17.8%; the most frequent AEs were abdominal pain managed with analgesics and acute post-RFA pancreatitis, which was managed conservatively in the majority of cases (22 out of 28 cases). The rate of severe AEs was 3.1%, and there were six cases of severe acute pancreatitis managed pharmacologically and endoscopically (only in one case, the patient underwent distal splenopancreasectomy for acute pancreatitis with pancreatic fistula, in which pancreatic stent placement failed) [58], one case of MPD injury, and two cases of MPD stenosis treated with pancreatic stent placement (and in one case, also biliary stent placement). Furthermore, Marx et al. [33] reported a single case of death of a 97-year-old patient, who developed fever and abdominal pain two weeks after the procedure and was subsequently diagnosed with retrogastric collection; the patient refused to undergo interventional maneuvers and died after two weeks despite supportive care.

Of 46 PCNs treated with EUS-RFA, the mean number of sessions/lesion was 1.03 (1.3 w.v.) for a total of 47.38 sessions (59.8 w.v.) (again, data from two studies were not taken into account due to the above-mentioned limitations [23,53]). The total number of AEs was 11 (total AE rate of 23.2%, 18.4% w.v.), with a single severe adverse event (2.1%, se usi ponderata 1.7%), namely a perforation of a jejunal loop that was treated surgically. The patient had been treated for an 18 mm IPMN of the uncinate process [17].

Of 153 PDAC lesions treated with EUS-RFA, the total number of sessions was 275.4 (306 w.v.), and the mean number of sessions/lesion was 1.8 (2.0 w.v.). The total number of adverse events was forty-eight (total AEs rate of 17.4% or, 15.7% w.v.), with six severe adverse events (2.2%, or 2.0% w.v.), namely, six cases of jaundice treated with endoscopic biliary prosthesis placement. In addition to the studies already mentioned, data from Testoni et al. [70] and Jiang et al. [72] were also excluded as they did not report data on complications.

With regard to patients with PMs, in particular, from clear-cell renal cell carcinoma, the available data are limited; Chanez et al. [79] reported two severe AEs in the only two patients treated with tyrosine kinase inhibitors (TKIs) at the time as EUS-RFA. In the first case, they reported the development of a duodenal abscess, and in the second case, in a patient with a biliary stent, the development of a hepatic abscess.

## 6. Conclusions

In conclusion, the advent of EUS has opened up new local therapeutic options for pancreatic lesions, and among these, radiofrequency ablation is one of those on which experts’ interest is focused. The EUS-RFA technique is feasible, repeatable, and safe, and the adverse events reported are mild in most cases, or, in cases of severe complications, they have almost always been successfully managed with endoscopic or surgical intervention. A standardized protocol for the EUS-RFA technique is not yet defined in relation to the device and the type of tissue and lesion to be treated. Furthermore, the literature data are confined to studies with small sample sizes and short follow-ups; the most encouraging data concern the application of EUS-RFA in pNETs, particularly in F-pNETs where it is aimed to be curative. The role of EUS-RFA still remains nebulous in PCLs, PDAC, and PMs, and prospective, randomized controlled studies are needed to answer the open questions on this topic.

## Figures and Tables

**Table 1:** Summary of developing indications, patient’s characteristics (considering MDT discussion), and mean lesion size reported in the available literature *.

	Indications	Characteristics of Patients	Mean Lesion Size (mm)
**pNETs**	Treatment for F-pNETs (insulinoma)	Elderly, unfit, or refusing surgery	13.8
	Treatment for NF-pNETs (G1/G2 grading)	Elderly, unfit, or refusing surgery/surveillance	15.2
	Combined treatment (surgery + EUS-RFA) in multifocal pNETs in MEN	MDT discussion	-
**PCLs**	Treatment for PCLs	Elderly, unfit, or refusing surgery	34.2
	Ablation of mural nodules in IPMNs and MCNs	Elderly, unfit, or refusing surgery	
	Cytovolumetric reduction in symptoms of compression	Elderly, unfit, or refusing surgery	
**PDAC**	Local treatment for cytovolumetric reduction in combination or not with CT	Locally advanced PDAC, unfit for surgery or with local progression after the first line of CT, or unfit for CT Metastatic PDAC	39.5
**PMs**	Local treatment for oligometastatic disease with PM	Stable oligometastatic disease, elderly, unfit, or refusing surgery	17.5

* The average size values correspond to the weighted average of the values given in Table 2, Table 3, Table 4, Table 5 and Table 6. CT: chemotherapy; IPMNs: intraductal papillary mucinous neoplasms; MCNs: mucinous cystic neoplasms; MDT: multidisciplinary team; MEN: multiple endocrine neoplasia; PCLs: pancreatic cystic lesions; PDAC: pancreatic ductal adenocarcinoma; PM: pancreatic metastasis; pNETs: pancreatic neuroendoscrine tumors.

**Table 3:** Results from studies reporting EUS-RFA on NF-pNETs *.

Author, Year	Study Design	N. of Patients/N. of NF-pNET	Tumor Size (Mean, mm) and Location **	Device	N. of RFA Sessions/Lesion **	Mean Time of Application (s)/Mean N. of Applications **	Technical Success (%) **	Follow-Up (Months) **	Radiologic Response
**Napoléon [23], 2023**	Retrospective, multicenter	NR/48	15 NR	EUSRA™, TaeWoong (50 W)	1.12	25/3	97	13	CR: 33/48 (71.7%) PR: 12/48 (26.1%) NoR: 1/48 (2.2%) Missing data 2
**Rizzatti [38],** **2023**	Prospective, multicenter	32/32	16.8 Head: 8 Body: 15 Tail: 9	EUSRA™, TaeWoong (50 W)	1.25	NR	100	12	CR: 24/24 (100%) Missing data 8
**Figueiredo Ferreira [39], 2022**	Prospective, multicenter	9/10	14.4 NR	EUSRA™, TaeWoong (50 W)	0.86	NR/3	100	9.6	CR: 6/11 (54.6%) PR: 3/11 (27.2%) NoR 2/11 (18.2%) Results F-pNET+ NF-pNET
**Marx [58], ** **2022**	Prospective, multicenter	27/27	14 Head: 8 Body: 8 Tail: 11	EUSRA™, TaeWoong (30–50 W)	1.15	10–12/1–5	100	15.7	CR: 25/27 (92.6%) NoR: 2/27 (7.4%)
**Younis [42], 2022**	Prospective, monocenter	6/6	8.9 Head: 2 Body: 4	EUSRA™, TaeWoong (50 W)	1	NR/2.7	100	7	CR: 4/6 (66.7%) NoR: 2/6 (33.3%)
**Lesmana [59], 2022–2024**	Case report	1/1	35 Head	EUSRA™, TaeWoong	1	NR	100	36	CR of the solid part, non-significant shrinkage of the cystic part
**De Nucci [44], 2020**	Prospective, monocenter	5/6	16 Head: 3 Body: 2 Tail: 2	EUSRA™, TaeWoong (20 W)	0.83	15–25/2.4	100	12	CR: 5/5 (100%)
**Barthet [17,21], ** **2019–2021**	Prospective, multicenter	12/14	13.4 Head: 3 Body: 6 Tail: 5	EUSRA™, TaeWoong (50 W)	NR	20–45/NR	100	45.6	CR: 12/14 (85.7%) NoR: 2/14 (14.3%) Including 1 late recurrence
**Oleinikov [50],** **2019**	Retrospective, multicenter	11/16	14.2 Head: 8 Body: 6 Tail: 2	EUSRA™, TaeWoong (10–50 W)	0.69	5–12/3–10	100	8.9	CR: 8/11 (72.7%) PR: 1/11 (9.1%) Missing data: 2
**Choi [13,27], ** **2018 and 2020**	Prospective, monocenter	13/13	18.1 Head: 5 Body: 7 Tail: 1	EUSRA™, TaeWoong (50 W)	1.69	NR/4.77	100	28	CR: 9/13 (69.2%) PR: 3/13 (23.1%) NoR: 1/13 (7.7%)
**Pai [11], ** **2015**	Prospective, multicenter	2/2	27.5 Head Body	Habib™ EUS RFA (5–25 W)	1.5	90–120/5	100	3–6	NR Change in vascularity
**Armellini [60], 2015**	Case report	1/1	20 Tail	EUSRA™, TaeWoong	1	NR	100	NR	CR
**Rossi [10], ** **2014**	Prospective, monocenter	1/1	9 Head	Habib™ EUS RFA (10–15 W)	1	360/1	100	34	CR

* Table legend: N. of RFA sessions/lesion expresses the ratio of the number of total procedures to the total number of pancreatic lesions. If equal to 1, the number of procedures is equal to the number of lesions; if >1, it indicates that some lesions are subjected to more than one session; if <1, it indicates that more lesions in the same patient are treated in the same session. Mean time of application (s) indicates the mean time per single application, expressed in seconds, to achieve the desired effect from ablation. Mean N. of applications indicates the mean number of applications needed to obtain complete treatment of the mass. CR: complete response; NoR: no response; NR: not reported; PR: partial response. ** In studies involving more than one type of pancreatic lesion, the overall data were reported when it was not possible to extract the specific data per lesion group.

**Table 4:** Results from studies reporting EUS-RFA on PCLs *.

Author, Year	Study Design	N. of Patients and Types of PCLs	Tumor Size (Mean, mm) and Location **	Device	N. of RFA Sessions/Lesion **	Mean Time of Application (s)/Mean N. of Applications **	Technical Success (%) **	Follow-Up (Months) **	Radiologic Response
**Napoléon [23], 2023**	Retrospective, multicenter	11 - 10 IPMNs with mural nodules - 1 SPT	29 (IPMN) 9 (SPT) NR	EUSRA™, TaeWoong (50 W)	1.12	25/3	97	13	CR: 5/10 (62.5%) PR: 3/10 (37.5%) Missing data: 1
**Younis [42], 2022**	Prospective, monocenter	5 - 4 IPMNs with worrisome features/mural nodules - 1 MCN	36 Head: 3 Body: 2	EUSRA™, TaeWoong (50 W)	1.6	NR/4	100	13	CR: 3/5 (60%) PR: 1/5 (20%) NoR: 1/5 (20%)
**Oh [26],** **2021**	Retrospective, monocenter	13 SCNs	50 Head: 5 Body /Tail: 8	EUSRA™, TaeWoong	1.5	NR	100	9.21	CR: 0/13 (0%) PR: 8/13 (61.5%) NoR: 5/13 (38.5%)
**Barthet [17,21],** **2019–2021**	Prospective, multicenter	17 - 16 IPMNs - 1 MCN with 12 mural nodules	29.1 Head: 10 Body: 4 Tail: 3	EUSRA™, TaeWoong (50 W)	1.12	20–45/NR	100	42.6	CR: 6/15 (40%) PR: 4/15 (26.7%) NoR: 5/15 (33.3%) 100% disappearance of mural nodules
**Choi [13], ** **2018**	Prospective, monocenter	2 SPNs	21.5 Head: 1 Tail: 1	EUSRA™, TaeWoong (50 W)	1	NR/4	100	13	CR: 1 (50%) PR: 1 (50%)
**Feng [16], ** **2018**	Case report	1 SCN	35 Tail	Habib™ EUS RFA + lauromacrogol injection after 2 months	1	120/3	100	3	NoR after 2 months, CR with combined therapy
**Thosani [53], 2018**	Retrospective, multicenter	1 SCN	23 NR	EUSRA™, TaeWoong	1.6	NR/4.6	100	5	NR
**Goyal [54],** **2017**	Case series	2 MCNs	20.5 Body: 1 Tail: 1	Habib™ EUS RFA (10 W)	1	120/3–5	100	NR	NR
**Pai [11], ** **2015**	Prospective, multicenter	6 - 4 MCNs - 1 IPMN - 1 Microcystic SCN	36.5 NR	Habib™ EUS RFA (5–25 W)	1	90–120/4.2	100	3–6	CR: 2/6 (33.3%) PR: 3/6 (50%) NoR: 1/6 (16.7%)

* Table legend: N. of RFA sessions/lesion expresses the ratio of the number of total procedures to the total number of pancreatic lesions. If equal to 1, the number of procedures is equal to the number of lesions; if >1, it indicates that some lesions are subjected to more than one session; if <1, it indicates that more lesions in the same patient are treated in the same session. Mean time of application (s) indicates the mean time per single application, expressed in seconds, to achieve the desired effect from ablation. Mean N. of applications indicates the mean number of applications needed to obtain complete treatment of the mass. CR: complete response; NoR: no response; NR: not reported; PR: partial response. ** In studies involving more than one type of pancreatic lesion, the overall data were reported when it was not possible to extract the specific data per lesion group.

**Table 5:** Results from studies reporting EUS-RFA on PDAC *.

Author, Year	Study Design	N. of Patients	Tumor Size (Mean, mm) and Location **	Device	N. of RFA Sessions/Lesion **	Mean Time of Application (s)/Mean N. of Applications **	Technical Success (%) **	Median Post-RFA Survival (Months)	Follow-Up (Months) **
**Robles-Medandra [67], in press**	Retrospective, monocenter	26 locally advanced and metastatic PDAC	39.5 Head: 22 Body: 3 Tail: 1	EUSRA™, TaeWoong (50 W)	1.27	5–10/NR	100	7	16.5
**Napoléon [23], 2023**	Retrospective, multicenter	6 PDAC	27 NR	EUSRA™, TaeWoong (50 W)	1.12	25/3	97	NR	13
**Kongkam [68], 2023**	Prospective, monocenter	14 locally advanced and metastatic PDAC	59.7 NR	EUSRA™, TaeWoong (50 W)	2.5	NR/5.6	100	NR	6
**Figueiredo Ferreira [39], 2022**	Prospective, multicenter	1 PDAC	14.4 NR	EUSRA™, TaeWoong (50 W)	0.86	NR/3	100	NR	9.5
**Oh [66],** **2022**	Retrospective, monocenter	22 locally advanced and metastatic PDAC	38 Head: 14 Body: 4 Tail: 3	EUSRA™, TaeWoong (50 W)	5	NR	100	Overall survival 24.03	21.23
**Thosani [69], 2022**	Prospective, monocenter	10 locally advanced and metastatic PDAC	39.2 Head: 4 Body: 4 Tail: 2	Habib™ EUS RFA (10–15 W)	2.2	NR	100	13.4	>30
**Testoni [70], 2022**	Retrospective, monocenter	13 locally advanced PDAC	42.3 Head: 8 Body/Tail: 5	HTP^®^ Probe, ERBE (18 W, 650 psi)	>1	125/NR	100	7	NR
**Lawerence [71], 2022**	Case series	2 locally advanced PDAC	NR	Habib™ EUS RFA (10 W)	3	120/NR	100	NR	NR
**Testoni [20], 2021**	Randomized controlled trial, Phase II	17 locally advanced and borderline resectable PDAC	47.1 Head: 10 Body /Tail: 7	HTP^®^ Probe, ERBE (18 W, 650 psi)	1	NR	94.1	13	45.4
**Jiang [72],** **2021**	Prospective, monocenter	8 locally advanced PDAC	46.9 Head: 5 Body: 2 Tail: 1	Habib™ EUS RFA (5 W)	1	95.3/2.4	100	10.7	NR
**Wang [73], ** **2021**	Retrospective, monocenter	11 locally advanced and metastatic PDAC	28 Head: 4 Body: 6 Tail: 1	Habib™ EUS RFA (5–10 W)	2.36	NR/3	100	5.2	12
**Ligresti [74], 2019**	Case report	1 resectable PDAC (unfit for surgery)	15 Body	EUSRA™, TaeWoong (20 W)	1	NR/2	100	NR	12
**Crinò [7], ** **2018**	Retrospective, monocenter	7 locally advanced PDAC	36 Head: 4 Body: 3	EUSRA™, TaeWoong (30 W)	1	58/1.5	100	NR	4.3
**Scopelliti [8], 2018**	Prospective, monocenter	10 locally advanced PDAC	49.2 Head: 4 Body: 6	EUSRA™, TaeWoong (20–30 W)	1	270/1.4	100	NR	1
**Thosani [53], 2018**	Retrospective, multicenter	10	23 NR	NR	1.6	NR/4.6	100	NR	5
**Goyal [54],** **2017**	Case series	2	26.5 Head: 2	Habib™ EUS RFA (10 W)	1	120/3–5	100	NR	NR
**Song [14], ** **2016**	Prospective, monocenter	6 locally advanced and metastatic PDAC	38 Head: 4 Body: 2	EUSRA™, TaeWoong (20–50 W)	1.33	10	100	NR	4.2
**Wang [75], ** **2013**	Case series	3	37.3	Habib™ EUS RFA (10–15 W)	1.67	120/NR	100	NR	1.6
**Arcidiacono [19], 2012**	Prospective, multicenter	22 locally advanced PDAC	35.7 Head: 13 Body: 9	HTP^®^ Probe, ERBE (18 W, 650 psi)	1	107/NR	72.8	6	NR

* Table legend: N. of RFA sessions/lesion expresses the ratio of the number of total procedures to the total number of pancreatic lesions. If equal to 1, the number of procedures is equal to the number of lesions; if >1, it indicates that some lesions are subjected to more than one session; if <1, it indicates that more lesions in the same patient are treated in the same session. Mean time of application (s) indicates the mean time per single application, expressed in seconds, to achieve the desired effect from ablation. Mean N. of applications indicates the mean number of applications needed to obtain complete treatment of the mass. NR: not reported. ** In studies involving more than one type of pancreatic lesion, the overall data were reported when it was not possible to extract the specific data per lesion group.

**Table 6:** Results from studies reporting EUS-RFA on PMs *.

Author, Year	Study Design	N. of Patients/N. of PMs	Tumor size (Mean, mm) and Location **	Device	N. of RFA Sessions/Lesion **	Mean Time of Application (s)/Mean N. of Applications **	Technical Success (%) **	Follow-Up (Months) **	Radiological Response
**Vohra [80], ** **2023**	Case report	1/1 mRCC invading duodenal wall	34 Head	EUSRA™, TaeWoong (50 W)	3	NR	100	3	NR
**Napoléon [23], 2023**	Retrospective, multicenter	NR/23 mRCC and other PM	17 NR	EUSRA™, TaeWoong (50 W)	1.12	25/3	97	13	CR: 7/23 (30.4%) PR: 10/23 (43.5%) NoR: 6/23 (26.1%)
**Figueiredo Ferreira [39], 2022**	Prospective, multicenter	NR/10 mRCC and PM of lung cancer	14.4 NR	EUSRA™, TaeWoong (50 W)	0.86	NR/3	100	9.5	CR: 1/9 (11.1%) NoR: 8/9 (88.9%) Missing data: 1
**Chanez [79], 2021**	Prospective, monocenter	12/21 mRCC	17 Head: 11 Body: 6 Tail: 4	EUSRA™, TaeWoong (50 W)	1.24	30–60/1–3	100	27.7	CR: 6/15 (40%) PR: 5/15 (33.3%) NoR: 4/15 (26.7%) Missing data: 6
**Biasutto [78], 2020**	Prospective, monocenter	3/4 mRCC	26 Head: 3 Body /Tail: 1	EUSRA™, TaeWoong (30 W)	NR	NR	100	3	NR
**Crinò [7], ** **2018**	Retrospective, monocenter	1/1 mRCC	22 Head	EUSRA™, TaeWoong (30 W)	1	55/1	100	3	PR

* Table legend: N. of RFA sessions/lesion expresses the ratio of the number of total procedures in relation to the total number of pancreatic lesions. If equal to 1, the number of procedures is equal to the number of lesions; if >1, it indicates that some lesions are subjected to more than one session; if <1, it indicates that more lesions in the same patient are treated in the same session. Mean time of application (s) indicates the mean time per single application, expressed in seconds, to achieve the desired effect from ablation. Mean N. of applications indicates the mean number of applications needed to obtain complete treatment of the mass. CR: complete response; NoR: no response; NR: not reported; PR: partial response. ** In studies involving more than one type of pancreatic lesion, the overall data were reported when it was not possible to extract the specific data per lesion group.

**Table 7:** Pooled data on AEs in EUS-RFA.

	AGREE I/II % of AEs (n)	AGREE III/IV/V % of AEs (n)
**pNETs** 325.9 EUS-RFA sessions *	5.8% abdominal pain (19) 6.8% acute pancreatitis (22) 0.6% post-procedural bleeding or upper GI ulcer (2) 0.6% spleen/gastric wall/peripancreatic hematoma (2) 0.3% diabetes (1) 0.6% other than GI AEs– fever (2) Total mild-moderate AEs 14.7%	1.8% acute pancreatitis (6) 0.3% abdominal collections (1, death) 0.3% MPD injury (1) 0.6% MPD stenosis (2) Total severe AEs 3.1%
**PCLs** 47.38 EUS-RFA sessions *	19.0% abdominal pain (9) 2.1% acute pancreatitis (1) Total mild-moderate AEs 21.1%	2.1% small bowel perforation or stricture (1) Total severe AEs 2.1%
**PDAC** 275.4 EUS-RFA sessions *	9.8% abdominal pain (27) 1.5% acute pancreatitis (4) 0.7% peripancreatic effusion (2) 0.7% ascites (2) 0.7% abdominal collections (2) 0.4% peritonitis (1) 0.4% spenoportal axis thrombosis (1) 0.4% post-procedural bleeding or upper GI ulcer (1) 0.4% small bowel perforation or stricture (1) 0.4% other than GI AEs– fever (1) Total mild-moderate AEs 15.3%	2.2% jaundice (6) Total severe AEs 2.2%
**PMs** 21.88 EUS-RFA sessions *	4.6% abdominal pain (1) Total mild-moderate AEs 4.6%	9.1% abdominal abscess (2) Total severe AEs 9.1%
**Not Specified [23,39,53] ^** 180 EUS-RFA sessions *	6.7% abdominal pain (12) 6.7% acute pancreatitis (12) 1.1% MPD injury (2) 2.2% MPD stenosis (4) 0.6% post-procedural bleeding or upper GI ulcer (1) 0.6% spleen/gastric wall/peripancreatic hematoma (1) 1.7% other than GI AEs—atrial fibrillation (1), fever (1), acute urine retention post-anesthesia (1) Total mild-moderate AEs 19.4%	1.1% acute pancreatitis (2) 0.6% MPD injury (1) Total severe AEs 1.7%

* In studies not reporting the N. of RFA sessions/lesion (NR), it was considered as 1. ^ Pooled data of three studies’ data in which AEs by lesion type cannot be derived.
